# Provider perspectives on telehealth for contraceptive care: “the provider isn’t going want to use it unless it’s easy to use”

**DOI:** 10.1186/s12978-026-02286-0

**Published:** 2026-02-28

**Authors:** Alejandra Alvarez, Sophie M. Morse, Yasaman Zia, Erica Somerson, Connie Folse, Kathryn Albergate Davis, Alison B. Comfort, Ghazaleh Moayedi, Suzan Goodman, Cynthia C. Harper

**Affiliations:** 1 Bixby Center for Global Reproductive Health, University of California San Francisco, 1001 Potrero Ave., UCSF, Box 0842, San Francisco, CA 94110 USA; 2 Department of Obstetrics, Gynecology and Reproductive Sciences, University of California San Francisco, 550 16Th Street, Box 0132, San Francisco, CA USA; 3 Philip R. Lee Institute for Health Policy Studies, University of California, San Francisco, 490 Illinois Street, Floor 7, Campus, Box 0936, San Francisco, CA 94158 USA; 4 Consultant, University of California, San Francisco, San Francisco, CA, United States; 5 Pegasus Health Justice Center, Dallas, TX USA; 6 Department of Family & Community Medicine, University of California San Francisco, 995 Potrero Ave, San Francisco, CA 94110 USA

**Keywords:** Telehealth, Contraceptive care, Provider perspective, Qualitative research, Implementation science

## Abstract

**Background:**

Telehealth services for contraception expanded significantly during the COVID-19 pandemic, but continued coverage for these services is uncertain. This study assessed providers’ recent experiences delivering contraceptive care via telehealth to improve these services.

**Methods:**

We conducted semi-structured qualitative interviews with healthcare practitioners in the U.S. providing contraceptive services (*N* = 41) from August 2022 to August 2024 to investigate telehealth practices for contraceptive care. We used thematic analysis to code the data and identify barriers and facilitators of telehealth use by providers. We identified themes using a modified Consolidated Framework for Implementation Research (CFIR) to assess factors that affected provision of telehealth contraceptive care.

**Results:**

We identified and mapped three main themes across CFIR domains: the incompatibility of telehealth with clinic systems and need for additional clinic support; varying preferences for in-person versus telehealth care; and the mixed impact of telehealth on patient privacy, agency, and rapport. Overall, participants enthusiastically discussed telehealth benefits for contraceptive care delivery. Some, however, limited their use of telehealth due to clinic requirements for Pap tests or onsite blood pressure readings for hormonal contraceptives. Many participants noted that judgment is needed to ensure privacy and caution in situations such as intimate partner violence and with certain patients such as adolescents.

**Conclusion:**

Results reflected the enduring benefits of telehealth for contraceptive care. The reported barriers highlight changes needed to efficiently expand the scope of telehealth services. Our findings point to the importance of prioritizing and investing in telehealth services to reach patients who otherwise face challenges completing in-person care. Additionally, these findings may inform ongoing policy debates about continued coverage for this innovative method of service delivery.

**Supplementary Information:**

The online version contains supplementary material available at 10.1186/s12978-026-02286-0.

## Abstract (plain language summary)

During the COVID-19 pandemic, many clinics began offering birth control through telehealth to help people to continue to receive contraceptive care. In this study, we aimed to assess healthcare practitioners’ experiences providing contraception services via telehealth. We asked about the challenges they have faced providing care via telehealth and the benefits. We conducted 41 interviews with doctors, clinicians, nurses, and health educators across the U.S. from August 2022 to August 2024. The three main themes providers discussed were: (1) challenges with incorporating telehealth into clinic systems and needed additional clinic support; (2) preferences by providers and patients for in-person care over telehealth; (3) telehealth impacted patient privacy, agency, and rapport in various ways. Overall, our findings showed that telehealth can improve access to contraception and increase patient privacy and agency, but clinics need better systems, updated guidance, and staff training to make it work well long-term.

**Supplementary Information:**

The online version contains supplementary material available at 10.1186/s12978-026-02286-0.

## Background

The expansion of telehealth, or use of phones, video, or computers to conduct remote medical or health education services [1], has reshaped U.S healthcare delivery. In response to the COVID-19 pandemic, the Department of Health and Human Services took steps to facilitate telehealth such as waiving Medicare telehealth service restrictions [2, 3]. Before 2020, most contraceptive providers had not used telemedicine or delivered clinical services using remote technology [4, 5]. However, during the pandemic, the offering of contraceptive care via telemedicine went from a rare occurrence, at 11% of providers, to the majority, at 79% across practice settings [6]. This increase is also reflected with telehealth under Medicare increasing from 5 million services provided during April-December 2019 compared to over 53 million services during April-December 2020 [3]. Use by people enrolled in Medicaid (who were able to use Medicaid in most states prior to the pandemic) has also increased [3]. Additionally, many patients switched to telehealth for prescriptions [7] including for self-administered subcutaneous depot-medroxyprogesterone acetate (DMPA-SQ) [8]. Many contraceptive providers surveyed during the pandemic considered telemedicine to be an appropriate method for delivering contraceptive counseling and felt it should continue [4]. In summary, telehealth use increased substantially after the start of the pandemic and policy changes such as waiving Medicare telehealth restrictions allowed many patients to access care through telehealth.

Telehealth offers care at the patient's convenience, making virtual visits accessible to patients in rural areas [9], as well as for patients with transportation difficulties, care-taking, and work responsibilities [10]. Research has also noted the benefits for continuation of care, as well as some limitations such as lack of personal connection between provider and patient [11]. Telehealth may facilitate autonomy for adolescents, allowing them to access contraceptive care without a parent or guardian present [12], although privacy and confidentiality concerns have been noted with virtual care [11].

Access to contraceptive care via telehealth, however, remains inequitable. Research has shown that young people and those facing financial and insurance gaps are less likely to use telemedicine for contraception [13, 14]. Regional differences exist, with people in the Midwest and South less likely to use telehealth [15]. Hispanic/Latinx people have also been documented to use less contraceptive telehealth visits than Black people [15]. Additional barriers include needing a phone or a computer with internet access [16].

Given an expansion of telemedicine services, along with persistent access inequities [17, 18], we examined providers’ experiences with delivering contraceptive care via telehealth to understand how to optimize this service delivery model. Building on past research of benefits and challenges to contraceptive provision during the early pandemic [11], we turn our focus to the late pandemic and post-pandemic period (2023–2024). Previous studies have predominantly focused on telemedicine for contraceptive care during the early stages of the pandemic (2020–2021) [4, 7, 11, 19]. Recognizing the practice and policy changes since the start of the pandemic when in-person health care was severely restricted, we aimed to assess provider experiences with delivering contraceptive care virtually in later years to identify factors that affected ongoing provision of telehealth contraceptive care. Post-COVID 19, providers had more experience with telehealth and in-person care had largely resumed, but how this mode of care can be sustained remains unknown. Using an implementation science approach grounded in the Consolidated Framework for Implementation Research (CFIR), we describe telehealth barriers and facilitators in contraceptive care delivery to shed light on how telehealth can be best integrated into contraceptive care moving forward. We use CFIR to look at the sustainability and sustainment of telehealth post-COVID, taking into account how the inner and outer settings and the nature of telehealth itself (captured in the implementation domain) affect ongoing telehealth use [20–22].

## Methods

We conducted semi-structured interviews with healthcare providers in 2022–2024 with the aim of evaluating their experiences providing contraceptive care through telehealth. Attendees of Continuing Medical Education (CME)-accredited contraceptive care trainings delivered by the University of California, San Francisco (UCSF) School of Medicine were invited to participate in a 45-min video interview on Zoom. We recruited the sample purposively, aiming for a range of providers and clinic staff (i.e. physicians, nurses, and health educators) across the country [23]. We included members of the healthcare team with direct patient interaction. Participants gave verbal informed consent and received a gift card for their time. Throughout the study, we refer to participants as “providers,” which we define broadly as people who provide clinical care, counseling or education for contraception, including clinic staff. The study was approved by the University of California San Francisco Institutional Review Board.

As part of a larger study on contraceptive care, the interview guide covered contraceptive counseling approaches, perceived patient care biases, and telemedicine’s role in contraceptive care. For this paper, we focused specifically on provider’s opinions and experiences delivering telemedicine, analyzing responses to the following topic guide questions: “*What do you think are some benefits and limitations of using telemedicine for contraceptive care?*”, “*In your experience, which patients/types of patients have benefitted from telemedicine*?” and “*Which patients may have benefited less from the expanded use of telemedicine*?” The interview guide was developed by the research team with input from our Advisory Board with a range of contraceptive experts that gives guidance on the provision of contraceptive care including updated modes. We piloted the interview guide with a limited number of participants for meaning and content of the questions, refined it, and piloted once more prior to study initiation. The interviewers were three cisgender women, who identify as Latinx, Jewish, and White (AA, ES, KAD). All interviewers received training from qualitative experts on conducting interviews and analyzing qualitative data. All interviews were recorded and transcribed.

### Data analysis

We collected and analyzed data concurrently, completing data collection once we achieved meaning saturation at 41 interviews [24]. We conducted thematic analysis to identify barriers and facilitators of current telehealth use by providers, using a codebook for data analysis [25]. The three interviewers (AA, ES, KAD) and an additional two team members (CF, YZ,) coded two interviews together to reconcile coding differences through discussion, grounded in a negotiated agreement approach [26]. We used a combination of deductive and inductive coding with a pre-established codebook; while most codes were established in advance based on the questions in the interview guide and the literature, we also contributed inductive codes based on emerging insights.

### Conceptual framework

We used the Consolidated Framework for Implementation Research (CFIR) to investigate telehealth implementation for contraceptive care delivery. This framework helps identify and systematically analyze potential barriers and facilitators to implementing interventions and is organized across five domains: 1) innovation, 2) outer setting, 3) inner setting, 4) individuals, and 5) implementation process [27, 28].

## Results

### Participant characteristics

Participants included physicians (*n* = 11), advanced practice clinicians (*n* = 22), nurses (*n* = 5), and health educators or social workers (*n* = 3). Participants were practicing in 18 states and Washington, DC, representing all regions of the US (Table 1). They were working at primary care practices, youth clinics serving adolescents and young adults, departments of health and family planning clinics and these facilities spanned urban, suburban and a few rural areas.

**Table 1:** Interview participant characteristics by provider type and region (*n* = 41)

	n	%
Provider Type		
Physician	11	27%
Advanced Practice Clinician	22	54%
Nurse	5	12%
Health Educator/Social Worker	3	7%
US Region^*^		
West	6	15%
Southwest	1	2%
Midwest	5	12%
Southeast	24	59%
Northeast	5	12%

^*^West: California, Oregon; Southwest: New Mexico; Midwest: Illinois, Indiana, Minnesota, Missouri; Southeast: Florida, Georgia, Louisiana, North Carolina, Oklahoma, Tennessee, Texas, Virginia; Northeast: New Jersey, New York, Washington, DC

### Themes organized by CFIR

**Fig. 1:**
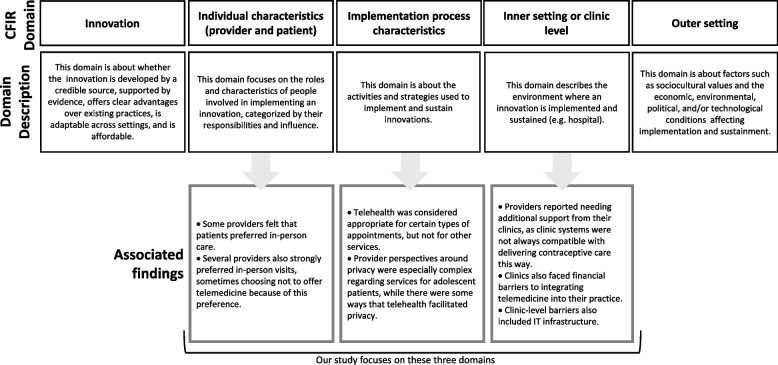
Findings presented by CFIR Domain

Based on interview content, our analysis centered on three CFIR domains: 1) inner setting or clinic level factors; 2) individual characteristics (provider and patient); and 3) implementation process characteristics. For clinic level factors, we analyzed characteristics of healthcare facilities where participants worked. For individual characteristics, we focused on providers as telehealth implementers and on their perceptions of patient telehealth experiences. For the implementation process, we explored how telehealth is used by providers and accessed by patients. We matched the themes that emerged in the thematic analysis with these CFIR domains, as shown in Fig. 1.

### Inner setting level/clinic level

#### Clinic support was needed for staff

Techniques and tools used for in-person care were sometimes reported as incompatible with telehealth. Some participants, such as the following college health center nurse practitioner, shared how educational materials are a vital part of contraceptive counseling and that the inability to show patients visual aids resulted in lower-quality visits.

“*If I’m counseling a patient in a room, I am using a lot of tools. I have...graphics with all these visual aids. I’m like, here’s what a ring looks like. And hold the IUD on my key chain.... I am not as comfortable using visual aids over telehealth. I’m sure the quality of the information... it’s just not as good*” (Nurse Practitioner, NJ).

Another nurse practitioner similarly reflected on the need for better digital tools,

“*The other issue is educational tools… I rely on that a lot in the clinic showing people models, showing pictures. And they can leave with those materials, so really to do it well, I think we would need to have something that goes with the patient electronically. Or something we can look together. And I certainly don’t have that capability right now. I would really like to see that piece.... we’re just such a small clinic that to develop that on our own, it’s really not realistic*” (Nurse Practitioner, CA).

Many participants reported not having the bandwidth to create or adapt electronic patient education tools to support contraceptive care provision via telehealth.

#### Telehealth incompatibility with clinic systems

In many cases, telehealth was incohesive with existing workflows and clinic protocols, which lacked a streamlined process for patients to complete pregnancy test or blood pressure check before appointments. A physician assistant explained “*if somebody called me today and said, ‘I want to do telehealth for new family planning’ I may be able to get the history, but I want to see them in person because I like to do a pregnancy test before I prescribe something*” (Physician Assistant, TX). Standard clinic-level policies for contraceptive screening and dispensing also limited the use of telehealth.

“*They have to come into the clinic to get a urine pregnancy test verification. Or a blood pressure check. I am only allowed to send 3 months of birth control if they haven’t had a normal blood pressure in the past 3 months. So that can be a barrier for those patients who really thought that they were going to get their full year prescription in one go.*” (Nurse Practitioner, MO).

There were frequently described discrepancies between methods available and the range offered via telehealth. For example, while self-injectable DMPA-SQ can be administered without a provider, some clinics did not initiate or teach this method via telehealth. One nurse practitioner (CA) explained, “*A whole category we’re not doing is DMPA-SQ, that people could do on their own.*” Some participants mentioned they could offer no-cost contraception to uninsured patients in the office, but not via telehealth.

“*As far as pills, a lot of our patients don’t have insurance so when they come to our clinic, we have pills there that are funded that we are able to give them*” (Nurse Practitioner, GA).

#### Workflow and Information Technology (IT) infrastructure impeded telehealth

Participants across clinic settings, including those working for health departments, expressed how telehealth must be convenient both for providers and patients to work. “*The provider isn’t going want to use it unless it’s easy to use*” (Registered Nurse, TN).

“*Telehealth was really time consuming in the beginning. Mainly because I wasn’t used to that way of seeing a patient, through a screen. And then there were a million questions built into this template that could take you forever to do. We only had 15 or 30 minute blocks for the whole entire encounter*” (Nurse Practitioner, LA).

Some participants described the tedious steps required to make telehealth appointments, which they felt discouraged people from accessing care this way. Other participants’ workloads limited their bandwidth to learn new systems, and they expressed needing support, around language barriers and interpreter services, especially among participants working at hospitals.

“*Interpretation services [make telehealth for contraceptive care more challenging]… especially if it’s a lesser used language. But we can have [the interpreter], the way our format works, like they can be on a video call with you. I just think it just adds a layer of complexity*” (Physician, TX).

#### Funding was lacking

Smaller clinic systems, especially those with limited resources, faced financial barriers to integrating telehealth. One nurse explained,

“*This clinic in particular was going through some growing pains, some financial issues so things were a little bit more chaotic, so priority in terms of elevating patient care [to include telehealth] was probably not there, it was just getting by each day*” (Registered Nurse, TN).

Some clinics or health systems lacked the resources to integrate telehealth into their practice, “*A big problem I see is more of a systems issue with healthcare being so fragmented. So you would love everyone to have that same telehealth capability, but small clinics might not have the funds to invest in it*” (Registered Nurse, TN). Thus, clinics with limited resources faced significant challenges to delivering care via telehealth.

### Individual patient and provider characteristics

Participants explained that many providers strongly preferred in-person visits, sometimes declining to offer telehealth. **“***Some people I work with might say they wouldn’t be comfortable prescribing birth control remotely”* (Nurse Practitioner, NY). Another shared,

“*The pandemic has encouraged people to think about health and how to get their services in a very different way. But I think that a lot of people still prefer that in-person type of communication and personally I like it better, in terms of just being able to connect with people****.**** I like that face to face versus just on a screen*” (Nurse Practitioner, TN).

Some participants felt this preference was influenced by their colleague’s age, with younger providers seeming more inclined to offer telehealth appointments,

*“I was working with a preceptor who was like in her 60 s and most of her patients, she never even suggested telehealth to them because she hated the telehealth system. She was like I don’t want to do telehealth, we’ll see you in 3 months. You’ll come into the clinic as it’s always been. And then recently started working also with a 30 year old provider and he was like, hey at your next visit if you’re comfortable we can do this via telehealth because you came in this time and all we have to do is go over your blood pressure logs. Obviously that’s a different scenario, but I’ve seen patients with okay I’ll just do that one on the phone then, and it will save me a trip into the clinic. So I’ve seen that really shift in patients through the provider being the one who offers it” (Registered Nurse, TN)*

Most participants reported the perception that patients overwhelmingly preferred in-person visits and the ability to access same-day contraceptive methods onsite,

“…*patients want to come in and have counseling and insertion or to leave with a prescription if that’s their chosen method same day… from a patient intentionality standpoint, they choose to do an in-person visit because they think they’ll be able to leave with the contraceptive same day*” (Registered Nurse, TN).

### Characteristics of the implementation process

#### Telehealth was considered appropriate for certain appointment types

Participants considered telehealth appropriate for certain types of services, but not for others, depending on the patient’s needs, which were not always clear prior to appointment. One nurse practitioner explained that telehealth visits “*work great for pill, patch, ring and the education piece of it. If the patient is not sure what they want to do but wants to talk about options, it’s fantastic…For Nexplanon or IUDs, they’re not great*” (Physician Assistant, MN). There was consensus that telehealth was appropriate for contraceptive counseling and for prescription refill visits.

“*[Telehealth is] vastly easier for somebody who just wants EC (emergency contraception) or oral contraception or a vaginal ring or something like that where I can just do a little chart review, and make sure they’re up to date on certain recommendations. Have they had a pap etc. Those kinds of visits are very easily streamlined by doing telemedicine*” (Physician Assistant, OR).

Participants explained a challenge with telehealth is when patients get lost in the follow-up process or don’t return for long-acting reversible contraceptive (LARC) (e.g., intrauterine device [IUD] or implant) placement. Some stressed challenges associated with offering contraceptive devices via telehealth when a symptom arose or when a patient wanted a LARC method. Additionally, a physical exam indication could emerge during a telehealth visit, requiring an additional in-person appointment.

“*But normally it’s just when other things come up and then I feel like okay, now you’re going to have to come back. If they were on site, then we would go ahead and evaluate those things and do that physical exam during that visit*” (Nurse Practitioner, OK).

#### Telehealth was both a barrier and facilitator for privacy and comfort

Participants’ reflections were mixed whether telehealth allowed patients to feel more comfortable by having a visit in a non-clinical space. They often considered telehealth a barrier to ensuring privacy and safety, especially for patients in vulnerable situations. Some shared that patients did not seem fully present or were reluctant to talk when they didn’t have privacy:

*“I’ll say are you in a private space, can I speak to you? Or sometimes they’ll go uh-huh. And I’ll say oh it sounds like maybe you don’t have privacy to talk about this. And they’ll be like uh-huh, I’ll say how about if I call you back, just say uh-huh when I hit the right time, 1 hour, 2 hours, tomorrow. So, I do think there are privacy concerns” ***(**Nurse Practitioner, NY*).*

Some mentioned it was more challenging to build trust with patients via telehealth, especially with patients from vulnerable populations (i.e. in substance use disorder treatment or foster care/group care settings). While telehealth allows contraceptive counseling initiation for some participants, in-person visits were preferred to build trust and obtain better information.

Opinions varied on whether telehealth or in-person visits promoted patients’ openness. While some participants noted pediatric patients “*open up a little bit more and talk about more things while we’re in person*” (Nurse Practitioner, NM), others noted that telehealth appointments aided in patients opening up more.

*“The privacy, like some people when they come in, they may be afraid of the White coat syndrome, and they really don’t want to be in a sterile clinic setting. But if they’re at home, they’re maybe more free to talk about it with the provider”* (Nurse Practitioner, TN).

Telehealth was perceived as beneficial in specific settings and challenging in others, depending on where patients connected to the appointment. Participants frequently mentioned that patients tried (sometimes unsuccessfully or with discomfort) to find a private space. In small town settings, however, telehealth offered privacy, especially for patients nervous about running into others in the clinic. A few participants expressed that telehealth was increasing access, noting that people from all over the state were accessing telehealth appointments with them,

*“I think patients also like being in their own home or their own safe space instead of having to come to the office and meet us for the first time. We’re also reaching patients outside of our small community. So we have people calling in from all over [the state] that just don’t have access. So it’s definitely increasing that access” *(Nurse Practitioner, IN).

#### There were concerns around privacy from parents for adolescents

Participants described frequent privacy concerns for adolescent patients unable to get away from family members to speak openly via telehealth. Workarounds for privacy during in-person visits were challenging for telehealth visits, with the potential for unintended consequences, as articulated by one nurse practitioner,

*“The mom was sitting there when they got the phone call with their positive STI. And then there was a big fight over that. So [sigh] yeah I think a lot of people live in close quarters with others”* (Nurse Practitioner, CA)*.*

Despite concerns about privacy, telehealth did afford some adolescent patient visits with increased confidentiality. Some participants mentioned telehealth as an alternative way to access care at school,

*“I feel like it has helped a lot of teens access [telehealth visits] during the day, where they may not be able to tell their parents that they need to be excused from school. So I have conducted numerous [visits] where people are in their school bathrooms”* (Nurse Midwife, IL).

#### Screening for safety for patients in vulnerable situations was complicated with telehealth

Some participants expressed how telehealth could act as a barrier to ensuring privacy and safety for patients in vulnerable situations. Participants expressed concern around the potential presence of a partner during telehealth contraceptive visits. Some described not being able to discern who was listening to patients potentially experiencing intimate partner violence (IPV),

*“When I have a patient with me in the room, I know who is in the room with me. With telehealth you don’t know all the other factors that are going on, and I think a lot of that has to do with my lens in terms of thinking what are the outside factors? Are they being pressured by a partner to do something they don’t want to do, and not being able to be in that space with somebody and read their non-verbals in a way they may be hiding if they’re able to just look at a screen”* (Nurse Practitioner, TN).

Some participants realized that IPV screening could be counterproductive in a telehealth visit and could jeopardize patient safety if a partner were listening. Relatedly, there was consensus that providers could only conduct an appropriate safety assessment if they knew who was present,

*“We also ask if a patient feels safe, that’s another regular question that we ask. If they feel like anybody is going to harm them. Whereas you know telemedicine either they would be with the person or they don’t see you and they don’t feel as comfortable talking to some stranger on the phone about that”* (Nurse Practitioner, GA).

Others worried a patient may not feel comfortable expressing contraceptive preferences openly in front of a partner, even without experiencing coercion or violence.

#### Rapport was diminished during telehealth visits

Participants agreed that developing rapport with patients was a key component of contraceptive care, and crucial for patients who may have experienced trauma or discrimination in health care settings. They described telehealth as impersonal, limiting nonverbal communication and patient rapport.

*“I really pick up on a lot of things as far as body language when people are there in person. And that’s still not the same with video either. So... kind of developing that relationship, works over the phone possibly a little better with video, probably the best in person”* (Nurse Practitioner, CA).

While video visits were described as superior to telephone visits for building rapport, participants overwhelmingly agreed that in-person visits were more productive and more easily facilitated rapport with patients.

“*Due to trauma, it is sometimes of greater comfort to first meet the medical provider that will ultimately be providing you your device. So telehealth is of course more impersonal. Sometimes when we have members of our community that are trans or nonbinary or coming from a traumatic background, I understand and want to provide that in office experience just because of the comfort of seeing and meeting them in person*” (Social Worker, TN).

“*Especially when talking about contraception, rapport and making your patient feel safe and having a sense of humor and all those things are so important... Because what I find is contraception is not a one and done…I think I do miss out a little bit on that kind of relationship*” (Nurse Practitioner, NJ).

## Discussion

This study filled gaps in knowledge on the barriers U.S. providers have faced providing telehealth contraceptive care in recent years. Clinic level barriers included compatibility issues, lack of infrastructure and support, all of which should be addressed on an institutional level. At the individual level, participants felt patients preferred in-person care, noting patients’ preference to leave with a method and feeling more comfortable speaking with a provider in-person. Regarding the implementation process, telehealth was seen by participants as suitable for certain appointments, including contraceptive counseling and refills, but less appropriate for services requiring vital signs or a pregnancy test for new prescriptions. Furthermore, for patients experiencing IPV or trauma, an in-person appointment can be important for rapport and safety because compromised privacy at home may allow for a partner to overhear responses, potentially causing harm or danger [29, 30].

Our findings illustrate how privacy could be supported by the use of telehealth, while in other cases, telehealth compromised patients privacy [11, 12, 31]. Some participants observed patient difficulties finding privacy to complete a telehealth visit while others reflected on how patients to complete visits on their own terms without others’ knowledge. Unlike previous research [11, 31], more participants cited telehealth as private or comfortable option for younger patients. While early pandemic research emphasized telehealth as a safety measure to prevent COVID spread, this did not come up in our current study [32] showing evolving telehealth concerns. Participants in this study reported how telehealth can facilitate privacy and autonomy, by allowing youth to access care without parental involvement [33, 34]. Participants also thought some youth seemed more comfortable receiving contraceptive care via telehealth, perhaps because they feel that telehealth care delivered via texting and calls is secure and private [35]. While previous research shows providers found telemedicine to help reaching youth while schools were closed [11], our study found that once schools were open again post-pandemic, telehealth remained helpful to reach youth while at school.

To best deliver contraceptive care and counseling to their patients, providers may need training on various aspects of healthcare delivery, such as up to date recommendations for telehealth contraceptive counseling and initiation. While assessing the likelihood of current pregnancy is recommended [36], it can be completed by history or home testing. Taking blood pressure is only recommended for patients with a history of hypertension in the last 3–5 years initiating or continuing combined hormonal contraception [44], not for non-hormonal or progestin-only methods, opening more opportunity for virtual contraceptive prescription [36]. The findings of this study also emphasize provider training needs in how delivery and scheduling platforms function. Contraceptive providers trained on telehealth have reported feeling more prepared to deliver care in this format compared to those who did not [37]. Providers needed additional support from their institutions to effectively provide telehealth, including training on methods that do not require in-person visits, such as the over-the-counter Opill®, approved in July 2023 [38], and self-injectable DMPA-SQ [8]. As telehealth has become more widely utilized, providers should be informed and trained on how to best provide care and counseling via telemedicine.

Our study also underscored a need to improve virtual language tools. Past research has shown that ease of communication promotes the use of telehealth [39], and Spanish speakers are less likely to choose telemedicine [10]. We found that providing telehealth for patients with less proficient English skills added another “layer of complexity” because interpretation services were not appropriately integrated into virtual visits, emphasizing the need to optimize clinic workflows for virtual care provided with an interpreter [40]. Additional training may improve provider comfort using interpretation services via telemedicine, which can be supported by an institutional investment in training [41].

While governmental support of telehealth was strong early in the pandemic, its coverage is now at risk. According to the Centers for Medicare and Medicaid Services, there are expected restrictions on the “scope of practitioners who can provide Medicare telehealth services” [42]. Medicaid restrictions on telehealth may further limit access to patients from lower socioeconomic backgrounds. Given the existing barriers to telehealth delivery and access, it is essential to protect Medicaid and Medicare coverage for telehealth to provide access to patients relying on telehealth for essential care, including contraception.

### Limitations

Our qualitative sample came from providers interested in contraceptive training, and who thus may have been more positive in their discussions of telehealth. Additionally, given our use of qualitative methods, we are unable to describe length or frequency of telehealth use among the sample of providers, which may be important for future studies utilizing quantitative methods such as surveys. We also must interpret participants’ observations about their patients with caution. While participants identified perceived patient perspectives, patients’ lived experiences may differ from or support our findings [43]. Future studies could interview patients on their experiences, including challenges and benefits, with receiving contraceptive care and counseling via telemedicine.

## Conclusion

Telehealth has expanded access to contraceptive counseling and care across the US, but many barriers continue to exist to its effective sustainment since the end of the COVID-19 pandemic. However, providers continue to face challenges implementing telehealth, highlighting necessary improvements to equitably reach patients and continue expanding its scope. Institutional investments to better integrate telehealth into clinic flow may facilitate an efficient and high quality experience for both patients and providers, but concerns around privacy and safety must be central to institutional policies. Prioritizing telehealth services and platforms and implementing staff trainings on the provision of the range of contraceptive method through telehealth should be part of clinic infrastructure improvement efforts. This will support providers, many of whom prefer in-person care, to better integrate telehealth into their practice. As telehealth coverage continues to be debated, our findings highlight how this care delivery model can be improved to reach patients and populations facing barriers to traditional in-person care.

## Supplementary Information

Supplementary Material 1.

## Data Availability

No datasets were generated or analysed during the current study.

## Abbreviations

CFIR: Consolidated Framework for Implementation Research
COVID-19: Coronavirus disease 2019
DMPA-SQ: Sub-Cutaneous Depo Medroxyprogesterone Acetate
EC: Emergency contraception
IPV: Intimate partner violence
IUD: Intrauterine device
LARC: Long-acting reversible contraceptive
