# Supplementary material for: Provider perspectives on telehealth for contraceptive care: “the provider isn’t going want to use it unless it’s easy to use”
Source: Reprod Health. 2026 Feb 28;23:70. doi: 10.1186/s12978-026-02286-0 (PMC13059397; doi:10.1186/s12978-026-02286-0)
Supplement: Supplementary file 1 — Supplementary Material 1. [file 12978_2026_2286_MOESM1_ESM.docx]

**Interview Guide**

*Interviewer: [Thank you for taking the time to speak with me.] We would like to hear about your experience providing contraceptive services (both in clinic as well as via telemedicine) and what might be most helpful in terms of training. There are no right or wrong answers. As a reminder, you can choose not to answer any questions or skip questions.*

1. First, can you tell me a bit about the clinic where you work and your own role, particularly as it relates to providing contraception?
   1. At your clinic, do you currently offer telemedicine?
      1. What types of contraceptive services is it used for?
      2. (If no) Why not, what are some barriers you have to providing care through telemedicine.
2. What do you think are some benefits and limitations of using telemedicine for contraceptive care?
   1. Are there certain situations or types of visits that make it more challenging? Can you give us some examples?
3. In your experience, which patients (or types of patients) have benefitted from telemedicine?
   1. Which patients may have benefited less from the expanded use of telemedicine?
   2. What might contribute to that?
   3. What about privacy concerns with telemedicine? Has that come up with your patients?
      1. What patient populations have been most affected by lack of privacy?
   4. What about issues of safety? Has that come up with your patients?
      1. What patient populations have been most affected by concerns of safety?
   5. What has been your experience with adolescents and young people and telemedicine for contraception?
   6. What are some ideas you have about how telemedicine could be changed or adapted to address those concerns around privacy and/or safety and/or access?

Now I’m going to ask you some questions about contraceptive care in general, including telemedicine and in-person care.

1. Could you tell me about any situations or interactions with patients that you have found particularly rewarding or fulfilling (in either in-person or telemedicine visits)? What are some examples of those interactions?
2. Could you describe a situation with a patient which prompted you to reflect on your own values or experiences and how they may impact the way you provide contraceptive care?
3. Can you tell me about any situations or interactions with patients that you have found to be frustrating or consistently challenging to deal with?
   1. What makes those interactions challenging?

Now I’d like to explore other challenges in providing care, including the impact of racism or discrimination on your patients.

1. In what ways have you seen racism impact your patients?
   1. In what ways have you seen other forms of bias or discrimination impact your patients?
      1. Have those observations impacted the way that you provide contraceptive care? If so, how?
      2. Have they impacted you in other ways?
   2. How about discrimination related to patients’ sexual or gender identity?
      1. How prepared do you feel to offer contraceptive care to LGBTQ+ patients?
         1. What has helped you feel prepared or capable?
         2. What else do you feel you need to be better prepared?
2. And now, thinking about your *own* experiences as a patient, have you ever felt that the care you received was biased? In what ways?
   1. How have your reflections on those experiences impacted the way that you provide care?

Interviewer: As a reminder, you attended a training on [DATE] that focused on how to *navigate common challenging contraceptive counseling scenarios*. The training also included a discussion of s*trategies for overcoming* ***implicit bias*** *and tools to help providers recognize and set aside personal values when counseling patients.*

1. Among the content that we covered during the training, what have you found to be most relevant to the work that you do?
   1. What are some of the reasons for that?
2. What are some content areas that are less applicable to you and your work?
   1. What changes to the course would make the training more relevant to your work?
   2. Are there any other topics you would want to see covered in a counseling training? What additional support around counseling would be helpful for you?
3. Can you describe for me any of the ways you think you have changed how you counsel your patients since participating in our training? If you have any specific examples, it would be great to hear them.
   1. Are there any counseling practices you would like to change or would like to see changed? What are some of the barriers to making those changes?
4. Have you participated in any other (non-BTP) trainings or workshops on implicit bias?
   1. [IF HAVE PARTICIPATED] What are some ways your perceptions of patients, or interactions with them, changed as a result?
   2. [IF HAVE NOT PARTICIPATED]: What are some reasons why you have not participated in these trainings?

*[Interviewer transition away from training to thinking about role as provider more broadly]*

1. How do you feel about your role as a provider of contraception for your patients after the recent SCOTUS ruling on abortion?
2. We’re nearing the end of the interview, but before we conclude, I would like to hear about anything you feel we missed.
   1. Are there any other things you’d like to share with me, that maybe I didn’t ask about, but you think are important for me to know?
   2. Do you have anything that you would like to ask me?

*Interviewer: Thank you for your time and candid answers. Your responses will help us to further improve our contraceptive training program. We are grateful for your participation.*
